# Cu_4.35_Cd_1.65_As_16_: the first polyarsenic compound in the Cu–Cd–As system

**DOI:** 10.1107/S1600536811042127

**Published:** 2011-10-22

**Authors:** Oliver Osters, Tom Nilges

**Affiliations:** a Technische Universität München, Department Chemie, Lichtenbergstrasse 4, 85747 Garching bei München, Germany

## Abstract

The first polyarsenic compound in the Cu–Cd–As system was obtained by solid-state reaction of the elements and has a refined composition of Cu_4.35 (2)_Cd_1.65 (2)_As_16_ (tetra­copper dicadmium hexa­deca­arsenide). It adopts the Cu_5_InP_16_ structure type. The asymmetric unit consists of one Cu site, a split Cu/Cd site and four As sites. The polyanionic structure can be described as being composed of As_6_ rings in chair conformations which are connected in the 1-, 2-, 4- and 5-positions. The resulting layers evolve along the *c* axis perpendicular to the *ab* plane. One Cu atom exhibits site symmetry 2 and is tetra­hedrally coordinated by four As atoms. The other Cu atom, representing the split site, and the corresponding Cd atom have different coordination spheres. While the Cu atom is tetra­hedrally coordinated by four As atoms, the Cd atom has a [3 + 1] coordination with a considerably longer Cd—As distance.

## Related literature

For Cu_5_InP_16_, see: Lange *et al.* (2008 ▶). For related polyphosphides, see: Pöttgen *et al.* (2006 ▶). For polyarsenides, see: Bauhofer *et al.* (1981 ▶); Jeitschko *et al.* (2000 ▶); Emmerling & Röhr (2002 ▶); Emmerling *et al.* (2004 ▶); Hönle *et al.* (2002 ▶). For binary Cu–Cd phases, see: Brandon *et al.* (1974 ▶); Kreiner & Schaepers (1997 ▶); von Heidenstamm *et al.* (1968 ▶). For related structures, see: Mansmann (1965 ▶); Clark & Range (1976 ▶). For crystallographic background, see: Becker & Coppens (1974 ▶).

## Experimental

### 

#### Crystal data

Cu_4.35_Cd_1.65_As_16_
*M* *_r_* = 1660.8
Monoclinic, 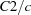

*a* = 11.8324 (6) Å
*b* = 10.4423 (4) Å
*c* = 8.0903 (4) Å
β = 110.480 (4)°
*V* = 936.44 (8) Å^3^
*Z* = 2
Mo *K*α radiation
μ = 34.73 mm^−1^
*T* = 293 K
0.030 × 0.020 × 0.004 mm

#### Data collection

Stoe IPDS 2T diffractometer
Absorption correction: numerical (*X-AREA*; Stoe & Cie, 2011 ▶) *T*_min_ = 0.205, *T*_max_ = 0.785
12811 measured reflections
1268 independent reflections
1113 reflections with *I* > 3σ(*I*)
*R*_int_ = 0.053

#### Refinement

*R*[*F* ^2^ > 2σ(*F* ^2^)] = 0.036
*wR*(*F* ^2^) = 0.072
*S* = 1.83
1268 reflections
56 parameters
Δρ_max_ = 1.50 e Å^−3^
Δρ_min_ = −1.67 e Å^−3^

### 

Data collection: *X-AREA* (Stoe & Cie, 2011 ▶); cell refinement: *X-AREA*; data reduction: *X-AREA*; program(s) used to solve structure: *Superflip* (Palatinus & Chapuis, 2007 ▶) embedded in *JANA2006* (Petřiček *et al.*, 2006 ▶); program(s) used to refine structure: *JANA2006*; molecular graphics: *DIAMOND* (Brandenburg & Putz, 2005 ▶); software used to prepare material for publication: *publCIF* (Westrip, 2010 ▶).

## Supplementary Material

Crystal structure: contains datablock(s) global, I. DOI: 10.1107/S1600536811042127/wm2539sup1.cif

Structure factors: contains datablock(s) I. DOI: 10.1107/S1600536811042127/wm2539Isup2.hkl

Additional supplementary materials:  crystallographic information; 3D view; checkCIF report

## Figures and Tables

**Table 1:** Selected bond lengths (Å)

Cu1—As1	2.4254 (9)
Cu1—As3	2.3931 (9)
Cu2—As2	2.516 (5)
Cu2—As3	2.501 (5)
Cu2—As4^i^	2.589 (6)
Cu2—As4^ii^	2.524 (7)
Cd2—As2	2.856 (5)
Cd2—As3	2.516 (5)
Cd2—As4^i^	2.475 (5)
Cd2—As4^ii^	2.569 (6)

Symmetry codes: (i) 
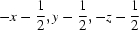
; (ii) 
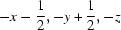
.

**Fig. 1.**
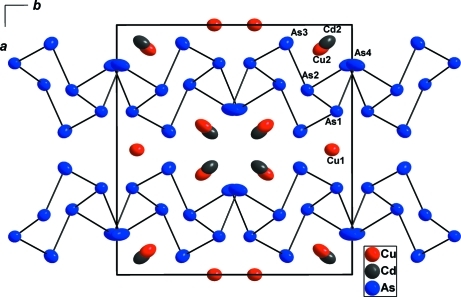
Crystal structure of Cu4.35 (2)Cd1.65 (2)As16, viewed along the c axis. Displacement ellipsoids are shown at the 90% probability level.

## supplementary crystallographic information

### Comment

Besides the plethora of known polyphosphides (Pöttgen *et al.*, 2006) only few polyarsenides are known up to date (Bauhofer *et al.*, 1981; Jeitschko *et al.*, 2000; Emmerling & Röhr, 2002; Emmerling  *et al.*, 2004; Hönle *et al.*, 2002).

The title compound Cu_4.35 (2)_Cd_1.65 (2)_As_16_ is the first representative of a polyarsenide adopting the Cu_5_InP_16_ structure type (Lange *et al.*, 2008). In accordance to the situation in Cu_5_InP_16_ where Cu and In are occupying the same site, a similar behavior is observed for the title compound, but here with a Cu/Cd split position. Mixing of Cu and Cd on one site is a common feature in intermetallic compounds and has been observed for instance for Cd_3_Cu_4_ (Kreiner & Schaepers, 1997) and Cd_8_Cu_5_ (von Heidenstamm *et al.*, 1968; Brandon *et al.*, 1974).

Cu—As distances in Cu_4.35 (2)_Cd_1.65 (2)_As_16_ range from 2.3931 (9) Å to 2.589 (6) Å and are comparable with the distances of 2.404 (1) Å to 2.590 (1) Å in Cu_3_As (Mansmann, 1965). The As—As distances in Cu_4.35 (2)_Cd_1.65 (2)_As_16_ are between 2.4242 (8) Å and 2.4644 (10) Å, in good accordance with the As—As distances in NdFe_4_As_12_ (2.428 Å - 2.499 Å) (Jeitschko *et al.*, 2000). Cd—As distances are present between 2.475 (5) Å and 2.856 (5) Å which is consistent with values found for CdAs (2.473 (2) Å - 2.868 (2) Å) (Clark & Range, 1976).

### Experimental

Cu_4.35 (2)_Cd_1.65 (2)_As_16_ was prepared by a solid state reaction from the elements Cu (ChemPur, shot, 99.999%), Cd (ChemPur, granules, 99.9999%) and As (ChemPur, pieces, 99.9999%). Arsenic was purified by sublimation in evacuated silica ampoules using a temperature gradient of 573 K to room temperature to separate As_2_O_3_ from the bulk-As and at 873 K to 573 K to sublimate As directly. The purified As was stored under protection gas atmosphere prior to use. The starting materials were reacted in stoichiometric amounts according the reported composition at 753 K for 7 days followed by a homogenization step by grinding. The procedure was repeated two times to finalize the formation of the title compound. Single crystals of suitable size could be separated from the bulk phase.

### Refinement

The highest peak is 0.99 Å away from As3 and the deepest hole is 0.81 Å away from As4. We have tested two different structure models to describe the Cu/Cd distribution in the title compound. In the first model, Cu and Cd were refined on one common position restricting the coordinates and displacement parameters while keeping an overall full occupancy. In the second model, the coordinates were not restricted, leading to a split position for Cu and Cd. Comparable to the first model the sum of occupancy factors of both split position were set to one. After an evaluation of the refinement results for both models we decided the second model for structure description due to better and more reliable displacement and statistical parameters.

### Crystal data

**Table:**

Cu_4.35_Cd_1.65_As_16_	*F*(000) = 1467
*M**_r_* = 1660.8	*D*_x_ = 5.888 Mg m^−^^3^
Monoclinic, *C*2/*c*	Mo *K*α radiation, λ = 0.71073 Å
Hall symbol: -C 2yc	Cell parameters from 12419 reflections
*a* = 11.8324 (6) Å	θ = 3.7–29.7°
*b* = 10.4423 (4) Å	µ = 34.73 mm^−^^1^
*c* = 8.0903 (4) Å	*T* = 293 K
β = 110.480 (4)°	Plate, black
*V* = 936.44 (8) Å^3^	0.03 × 0.02 × 0.004 mm
*Z* = 2	

### Data collection

**Table:**

Stoe IPDS 2T diffractometer	1268 independent reflections
Radiation source: X-ray tube	1113 reflections with *I* > 3σ(*I*)
plane graphite	*R*_int_ = 0.053
Detector resolution: 6.67 pixels mm^-1^	θ_max_ = 29.3°, θ_min_ = 3.7°
ω scans	*h* = −16→16
Absorption correction: numerical (*X-AREA*; Stoe & Cie, 2011)	*k* = −14→14
*T*_min_ = 0.205, *T*_max_ = 0.785	*l* = −11→11
12811 measured reflections	

### Refinement

**Table:**

Refinement on *F*^2^	6 constraints
*R*[*F*^2^ > 2σ(*F*^2^)] = 0.036	Weighting scheme based on measured s.u.'s *w* = 1/[σ^2^(*I*) + 0.0004*I*^2^]
*wR*(*F*^2^) = 0.072	(Δ/σ)_max_ = 0.007
*S* = 1.83	Δρ_max_ = 1.50 e Å^−^^3^
1268 reflections	Δρ_min_ = −1.67 e Å^−^^3^
56 parameters	Extinction correction: B-C type 1 Gaussian isotropic (Becker & Coppens, 1974)
0 restraints	Extinction coefficient: 0.021 (2)

### Fractional atomic coordinates and isotropic or equivalent isotropic displacement parameters (Å^2^)

**Table:**

	*x*	*y*	*z*	*U*_iso_*/*U*_eq_	Occ. (<1)
Cu1	0	0.41450 (10)	0.25	0.0156 (3)	
Cu2	−0.0942 (5)	0.1309 (5)	−0.0886 (9)	0.0198 (9)	0.587 (6)
Cd2	−0.0713 (4)	0.1064 (5)	−0.0867 (7)	0.0198 (9)	0.413 (6)
As1	−0.15337 (5)	0.56586 (5)	0.08461 (8)	0.01309 (19)	
As2	−0.23875 (5)	0.30964 (6)	−0.22826 (8)	0.01406 (19)	
As3	0.07426 (6)	0.27955 (6)	0.07165 (9)	0.0171 (2)	
As4	−0.33882 (6)	0.48506 (7)	−0.13511 (9)	0.0232 (2)	

### Atomic displacement parameters (Å^2^)

**Table:**

	*U*^11^	*U*^22^	*U*^33^	*U*^12^	*U*^13^	*U*^23^
Cu1	0.0132 (5)	0.0182 (5)	0.0157 (5)	0	0.0055 (4)	0
Cu2	0.0171 (16)	0.0237 (16)	0.0194 (4)	−0.0067 (9)	0.0076 (10)	−0.0062 (10)
Cd2	0.0171 (16)	0.0237 (16)	0.0194 (4)	−0.0067 (9)	0.0076 (10)	−0.0062 (10)
As1	0.0115 (3)	0.0139 (3)	0.0130 (3)	−0.0003 (2)	0.0033 (2)	0.0001 (2)
As2	0.0126 (3)	0.0143 (3)	0.0143 (3)	0.0015 (2)	0.0034 (2)	0.0016 (2)
As3	0.0153 (3)	0.0172 (3)	0.0198 (3)	0.0025 (2)	0.0074 (2)	0.0046 (2)
As4	0.0128 (3)	0.0296 (3)	0.0265 (4)	−0.0031 (3)	0.0061 (3)	−0.0155 (3)

### Geometric parameters (Å, °)

**Table:**

Cu1—As1	2.4254 (9)	Cd2—As2	2.856 (5)
Cu1—As1^i^	2.4254 (9)	Cd2—As3	2.516 (5)
Cu1—As3	2.3931 (9)	Cd2—As4^ii^	2.475 (5)
Cu1—As3^i^	2.3931 (9)	Cd2—As4^iii^	2.569 (6)
Cu2—Cd2	0.370 (8)	As1—As2^v^	2.4644 (10)
Cu2—As2	2.516 (5)	As1—As3^vi^	2.4307 (10)
Cu2—As3	2.501 (5)	As1—As4	2.4408 (8)
Cu2—As4^ii^	2.589 (6)	As2—As3^vii^	2.4242 (8)
Cu2—As4^iii^	2.524 (7)	As2—As4	2.4392 (10)
Cd2—Cd2^iv^	2.845 (7)		
			
As1—Cu1—As1^i^	98.67 (4)	As3^vi^—As1—As4	105.22 (3)
As1—Cu1—As3	114.38 (2)	Cu2—As2—Cd2	3.1 (2)
As1—Cu1—As3^i^	110.77 (2)	Cu2—As2—As1^viii^	107.87 (17)
As1^i^—Cu1—As3	110.77 (2)	Cu2—As2—As3^vii^	109.44 (12)
As1^i^—Cu1—As3^i^	114.38 (2)	Cu2—As2—As4	138.19 (17)
As3—Cu1—As3^i^	107.85 (4)	Cd2—As2—As1^viii^	105.21 (13)
Cd2—Cu2—As2	155.1 (16)	Cd2—As2—As3^vii^	108.99 (10)
Cd2—Cu2—As3	88.1 (11)	Cd2—As2—As4	141.07 (12)
Cd2—Cu2—As4^ii^	68.2 (12)	As1^viii^—As2—As3^vii^	105.49 (3)
Cd2—Cu2—As4^iii^	92.7 (15)	As1^viii^—As2—As4	98.12 (3)
As2—Cu2—As3	93.76 (19)	As3^vii^—As2—As4	93.81 (3)
As2—Cu2—As4^ii^	95.40 (19)	Cu1—As3—Cu2	106.49 (17)
As2—Cu2—As4^iii^	110.1 (3)	Cu1—As3—Cd2	113.57 (14)
As3—Cu2—As4^ii^	139.5 (3)	Cu1—As3—As1^vi^	102.24 (3)
As3—Cu2—As4^iii^	108.6 (2)	Cu1—As3—As2^ix^	105.39 (3)
As4^ii^—Cu2—As4^iii^	105.0 (2)	Cu2—As3—Cd2	8.44 (18)
Cu2—Cd2—Cd2^iv^	149.6 (16)	Cu2—As3—As1^vi^	121.65 (17)
Cu2—Cd2—As2	21.8 (14)	Cu2—As3—As2^ix^	118.91 (13)
Cu2—Cd2—As3	83.5 (11)	Cd2—As3—As1^vi^	122.14 (14)
Cu2—Cd2—As4^ii^	103.9 (12)	Cd2—As3—As2^ix^	111.47 (11)
Cu2—Cd2—As4^iii^	79.0 (15)	As1^vi^—As3—As2^ix^	100.07 (3)
Cd2^iv^—Cd2—As2	170.7 (3)	Cu2^x^—As4—Cu2^iii^	145.40 (19)
Cd2^iv^—Cd2—As3	97.37 (17)	Cu2^x^—As4—Cd2^x^	7.97 (17)
Cd2^iv^—Cd2—As4^ii^	92.32 (19)	Cu2^x^—As4—Cd2^iii^	138.18 (18)
Cd2^iv^—Cd2—As4^iii^	71.60 (19)	Cu2^x^—As4—As1	110.54 (12)
As2—Cd2—As3	85.72 (15)	Cu2^x^—As4—As2	102.12 (16)
As2—Cd2—As4^ii^	89.91 (14)	Cu2^iii^—As4—Cd2^x^	138.99 (18)
As2—Cd2—As4^iii^	99.07 (19)	Cu2^iii^—As4—Cd2^iii^	8.26 (17)
As3—Cd2—As4^ii^	146.1 (3)	Cu2^iii^—As4—As1	94.09 (12)
As3—Cd2—As4^iii^	106.8 (2)	Cu2^iii^—As4—As2	99.75 (14)
As4^ii^—Cd2—As4^iii^	107.07 (19)	Cd2^x^—As4—Cd2^iii^	132.39 (17)
Cu1—As1—As2^v^	113.16 (3)	Cd2^x^—As4—As1	118.43 (12)
Cu1—As1—As3^vi^	111.70 (3)	Cd2^x^—As4—As2	101.77 (14)
Cu1—As1—As4	119.01 (3)	Cd2^iii^—As4—As1	96.10 (10)
As2^v^—As1—As3^vi^	106.52 (3)	Cd2^iii^—As4—As2	107.52 (12)
As2^v^—As1—As4	99.93 (3)	As1—As4—As2	94.29 (3)

Symmetry codes: (i) −*x*, *y*, −*z*+1/2; (ii) −*x*−1/2, *y*−1/2, −*z*−1/2; (iii) −*x*−1/2, −*y*+1/2, −*z*; (iv) −*x*, −*y*, −*z*; (v) *x*, −*y*+1, *z*+1/2; (vi) −*x*, −*y*+1, −*z*; (vii) *x*−1/2, −*y*+1/2, *z*−1/2; (viii) *x*, −*y*+1, *z*−1/2; (ix) *x*+1/2, −*y*+1/2, *z*+1/2; (x) −*x*−1/2, *y*+1/2, −*z*−1/2.
